# Improving management of student clinical placements: insights from activity theory

**DOI:** 10.1186/s12909-016-0747-5

**Published:** 2016-08-24

**Authors:** Maree O’Keefe, Victoria Wade, Sue McAllister, Ieva Stupans, Teresa Burgess

**Affiliations:** 1 Faculty of Health Sciences, The University of Adelaide, Adelaide, Australia; 2 Faculty of Health Sciences, Flinders University, Adelaide, Australia; 3 School of Health and Biomedical Sciences, RMIT University, Melbourne, Australia

**Keywords:** Clinical placements, Health, Students, Clinical teams, Learning, Activity theory

## Abstract

**Background:**

An approach to improve management of student clinical placements, the Building Teams for Quality Learning project, was trialed in three different health services. In a previous paper the authors explored in some detail the factors associated with considerable success of this approach at one of these services. In this paper, the authors extend this work with further analysis to determine if the more limited outcomes observed with participants at the other two services could be explained by application of activity theory and in particular the expansive learning cycle.

**Methods:**

Staff at three health services participated in the Building Teams for Quality Learning project: a dental clinic, a community aged care facility and a rural hospital. At each site a team of seven multi-disciplinary staff completed the project over 9 to 12 months (total 21 participants). Evaluation data were collected through interviews, focus groups and direct observation of staff and students. Following initial thematic analysis, further analysis was conducted to compare the processes and outcomes at each participating health service drawing on activity theory and the expansive learning cycle.

**Results:**

Fifty-one interview transcripts, 33 h of workplace observation and 31 sets of workshop field notes (from 36 h of workshops) were generated. All participants were individually supportive of, and committed to, high quality student learning experiences. As was observed with staff at the dental clinic, a number of potentially effective strategies were discussed at the aged care facility and the rural hospital workshops. However, participants in these two health services could not develop a successful implementation plan. The expansive learning cycle element of *modeling and testing new solutions* was not achieved and participants were unable, collectively to reassess and reinterpret the object of their activities.

**Conclusion:**

The application of activity theory and the expansive learning cycle assisted a deeper understanding of the differences in outcomes observed across the three groups of participants. This included identifying specific points in the expansive learning cycle at which the three groups diverged. These findings support some practical recommendations for health services that host student clinical placements.

## Background

In the course of their studies, health profession students are placed in many different clinical environments. Health service staff, in turn, are asked by universities to host placements for students from different disciplines and institutions, often simultaneously. In addition, students come into these health services with variable levels of proficiency and experience. For many reasons, staff working in health service environments vary in their ability to flexibly respond to this complexity [1].

A key challenge for health service staff is managing the competing demands of clinical service and student supervision [2–4]. When these two activities are in conflict due, for example, to high patient care workloads and time pressures, patient care will take priority [5, 6]. At a more subtle level, there may also be an impact on the selection of ‘suitable’ patients for student interactions, and even the extent to which students are actively engaged or merely observers in the clinical encounter [5, 6].

Increasing university enrolments into health care education programs and new models of health care delivery and funding have resulted in a greater diversity in clinical placement settings [7, 8]. Such changes increase the likelihood that students are supervised by less experienced clinicians and less likely to be active participants in health care delivery [7, 9]. A lack of health service staff familiarity with newer curriculum models and teaching approaches, and in some cases actual resistance to innovation can also adversely affect student-learning experiences [10].

Actively engaging students as participants in patient care contributes positively to student learning [11–13]. The extent to which students can be legitimate participants in health care delivery is influenced by the prevailing workplace culture including local interpretations of the work versus learning expectations of both staff and students [14–16]. A shared understanding of the purpose of the placement in both students and supervising clinicians is important [7]. There are also calls for the development of new ways to work to assist existing health services and their staff to manage student clinical supervision [7, 17].

In order to contemplate of new ways to work, there is a need to consider the nature and purpose of day-to-day activities. In an earlier study, the authors describe an approach to working with health service staff to improve management of student clinical placements, the Building Teams for Quality Learning project [18]. A key element of this approach is to consider ‘who is doing what and why’ to support student learning on clinical placements. This is followed by a comparison of the nature and purpose of existing daily activities with a set of aspirational daily activities that would better integrate students into the working environment. The final activity with participants involves the implementation of change in daily activities.

Building Teams for Quality Learning was trialed in three different health services with quite different outcomes. The authors have previously reported in detail on one health service, a dental clinic, in which staff did progress to successful implementation of a change in daily activities to better accommodate students on clinical placements [19]. Participating staff in the other two health services, an aged care service and a rural hospital, evaluated current activities and developed a set of aspirational plans. However, in these latter two groups, actual change was not achieved. With reference to activity theory a comparative analysis of three sites and further analysis of the dental clinical data was undertaken.

Activity theory considers the various activities people engage in to achieve specific outcomes or ‘objects’ [20–23]. These activities encompass individual personal objectives, interactions between the people, the organisation of work tasks, and the wider community of participants.

Activity theory was selected as a theoretical lens due to the accommodation of complex working environments such as health care settings with multiple activity systems. In addition, activity theory brings together considerations of the interactions of people, purpose and tools together with the concept of boundary objects as facilitators of change [24, 25]. The associated expansive learning cycle (Table 1) proposes that successful change involves a transformation in understanding or ‘expansion’ of the object of the activity [23].

**Table 1:** Sequence of learning actions associated with expansive learning cycle

1. Questioning/Need	
2. Analysis/Double bind	
3. Modeling the new solution/Breakthrough	
4. Examining and testing the new model/Adjustment, enrichment	
5. Implementing the new model/Resistance	
6. Reflecting on the process/Stabilisation	
7. Consolidating and generalizing the new practice	

Engestom Y, Sannino A. Studies of expansive learning: foundations, findings and future challenges. *Educ Res Rev* 2010; **5**:1-24

In particular, the theory of expansive learning describes a process whereby individuals come together to question their existing work activities and in so doing, begin to develop a more cohesive network. The early stages of this cycle involve the identification of a need for change and the associated constraints within existing activity system/s. A contradiction within the activity system can trigger this process [23]. Different expectations of health services and universities regarding student placement activities is one example of a contradiction that may lead to an expansive learning cycle occurring. A ‘breakthrough’ occurs when participants identify a new solution that challenges preexisting arrangements and understandings of the object of the activity. With successful completion of the cycle, the entire activity system moves to a new equilibrium.

At the dental clinic Building Teams for Quality Learning facilitated a transformational process. Staff moved beyond lamenting the need for change and cataloging the barriers, to trialing and then embedding new approaches to managing student clinical placements. Clinical placements became a more integrated part of clinical service delivery, with students treated more as team members rather than supernumeraries. In contrast, staff at the two other sites, the aged care facility and the rural hospital, became ‘stuck’ at the point of trying to implement their new ideas and a number of innovative solutions to current challenges stalled.

In this paper, the authors extend their previous work to determine if the more limited outcomes in the aged care facility and the rural hospital can also be explained by application of activity theory and the expansive learning cycle.

## Methods

The Building Teams for Quality Learning project was conducted between 2008 and 2010 in South Australia, Australia. Academic staff from each of the three Universities in the state collaborated in development and implementation. At that time there was concern that the number of clinical placements available to health profession students would be insufficient in the near future to cope with increasing enrolment numbers in medical, dental and nursing degree programs. Health service staff were also reporting increasing clinical workloads that they believed were compromising their capacity to supervise students on placements.

Participating health services were selected to ensure varying levels of complexity, location and discipline composition. Senior management was approached at three statewide organisations that hosted student clinical placements during the duration of the project. In each case the clinical supervision of students was identified as posing many challenges. The proposed activities were seen to offer value to the organisation and specific sites were nominated for participation. Individual staff were then approached and invited to participate. An action research method was used to enable refinement of the approach in sequential testing to maximise effectiveness [26, 27].

Each health service accepted students for clinical placements throughout the year, with different disciplines and universities represented at each service. Selection of participants varied in each service as is described below. Data was collected through interviews, focus groups and direct observation.

### Participants and site descriptions

Site A (dental clinic) was a small single-discipline public clinic in a rural area, delivering services in an area of high need, had long waiting lists, and patients frequently presenting with urgent conditions which needed attention, hence they were very busy. All staff were invited to participate. Seven staff of the nine clinic staff agreed to participate (three dentists, two dental therapists, one dental assistant, one manager). The team was mostly comprised of long serving staff and was reasonably tight knit. Senior students from one university who could deliver direct patient care under supervision were placed there for 2-week blocks of time. The team had autonomous control of the clinic organisation. There were clear rules and divisions of responsibility for patient care and student supervision with the dentists sharing sole responsibility for the students. Three contradictions were identified. The first was that students were positioned away from the main clinic area and often needed to wait for a dentist to become available to come and check their work, thus delaying patient care. The second was that the clinic booking system did not allow for students to have different amounts of time allocated to patient appointments to cater for different levels of student experience and clinical competence. The third contradiction was that in providing clinical care to clinic patients, the students had to interact with staff other than the supervising dentists, such as the dental therapist, who were unclear about their supervisory responsibility towards the students.

Site B (aged care facility) was a large multidisciplinary community aged care facility situated in an urban area. It was part of a much larger aged care organisation. At the request of the local facility manager, the members of one established clinical team were invited to participate. Seven staff participated (three nurses, one carer, one physiotherapist, one manager, one lifestyle coordinator). There were a large number of staff within the facility who were not invited to participate. The service was highly structured, and had many long-standing staff who were very busy and had honed their working day to a high level of efficiency: the ‘well oiled machine’. Students from a number of different institutions including three universities were hosted for a variety of clinical placements. These students included all levels of nursing, senior allied health, aged care workers, and high school students. Most student clinical placements were of short duration (several weeks). Although there were clear rules and divisions of responsibility for resident care for staff, responsibility for student supervision was organised on a more ad hoc basis and was quite variable. The culture of the organisation was strongly hierarchical and inflexible with students rostered to ‘work’ standard shifts alongside the staff. Two contradictions were identified. Firstly, the students had logbooks that specified their required learning outcomes, which did not always align with the service expectations of them for work. Secondly, students were not inducted into the workplace in the same ways as staff even though the students had resident care responsibilities.

Site C (rural hospital) was a medium sized rural hospital serving a large hinterland. It contained a wide range of disciplines, and staff reported chronic workforce shortages, organisational stress, high staff turnover, and lack of integration of many key functions including the management of student supervision. Seven staff participated (three nurses, one midwife, one doctor, one manager, one ward clerk). These participants were nominated by the local hospital manager and were drawn from across the hospital. Medical, nursing and allied health students from three different universities were being hosted for clinical placements, for periods ranging from a few weeks to several months. As with the aged care facility, the team at the rural hospital was part of a larger staff at the institution. In contrast to the aged care facility, the hospital presented itself as being in a state of chaotic dysfunction at an organisational level. A clear contradiction arising from this situation for individual staff was that student supervision was expected to occur whilst they were functioning in crisis mode.

### Data collection

Building Teams for Quality Learning is a professional development approach to build capacity for collective action among a group of health professionals to address local issues impacting on the management of student clinical placements. Members of the study team engaged with project participants in workshops and during the collection of data as described below.

The project was implemented sequentially at 6-month intervals (commencing with Site A and concluding with Site C). At each site the period of active engagement with participants was between 9 and 12 months. The project started with baseline individual participant interviews and workplace field observations. Students placed at the site were also interviewed and observed. Participating staff then completed an activity that focused on their individual preferences for managing student learning in the workplace, and attended a one-day workshop. At the workshop participants shared their own preferences, explored complementarity with their colleagues and identified any gaps or overlaps in preferences. A plan to improve management of student clinical placements was developed by the participants for implementation over the subsequent 6-months with a particular focus on contradictions that had been identified in currently practices and processes.

A second round of participant interviews and workplace field observations was undertaken 6 months later. Following this second round of data collection, participants completed a 360-degree style evaluation of their management of student clinical placements and attended a full day workshop to review their progress on implementation of their improvement plan. Finally, an independent evaluator conducted an exit group interview with health service staff and students 2 to 3 months after the second workshop.

Participant interviews were semi-structured with an identical schedule used at all three sites [19]. The baseline interview focussed on participant perceptions of their roles and responsibilities as team members for managing student learning activities. The 6-month interview focussed on changes in team functioning. In the exit interview, participant perceptions, expectations and experiences during the project were discussed. Two researchers conducted the interviews and the workplace observations, with interviews and observations at each site conducted by the same researcher. All interviews were digitally recorded with de-identified transcriptions produced. Detailed workplace field observations focussed on student activity and interaction with health service staff. In addition, members of the research team recorded field notes at workshops on participant interaction and engagement.

### Data analysis

All data transcripts were entered into NVivo software v8 [28]. Following initial open coding, the first round of qualitative analysis categorised the impact of the projects at the three sites, according to Strauss and Corbin’s conditional/consequential matrix [29]. The analysis began with the individuals who participated in the project, moving outward to the interaction of the clinical team with students and the context of the health care organisation as a whole. An assessment of overall resistance to change was added to the matrix as it was judged to be pertinent to the final outcome. Triangulated comparisons were made between different data sources to strengthen the credibility of the findings. Any inconsistencies were discussed among project team members with reference back to the original transcript data as required to determine a consensus view as to the appropriate recording of such a finding.

A second round of analysis was conducted based on expansive learning as a staged model of organisational change [23]. With a focus on elucidating the processes of change, the main goal of this analysis was to develop a better understanding and appreciation of the variations in overall outcomes observed at each site.

## Results

Fifty-one interview transcripts, 33 h of workplace observation and 31 sets of workshop field notes (from 36 h of workshops) were generated (Table 2). Following the initial data analysis, it was apparent that most participants reported a personal benefit arising from an increased self-awareness through completing the individual personal work preference profiles, and an enjoyment of the personal interactions with their colleagues. Moving beyond the individual level to team functioning, outcomes became more variable across the sites. As has been previously reported, staff at the dental clinic were highly engaged with the project activities. Responding to the contradictions identified in the workshops relating to student location, student bookings and supervision, the physical layout, workflow, supervision processes and clinical workloads were all altered. Staff developed greater team cohesion and expanded their understanding of responsibility for student supervision from a single designated supervisor to include the whole multi-disciplinary team.

**Table 2:** Data sources by site and participants

Data sources	A Dental clinic	B Aged care	C Rural hospital
Workplace observation (Hours)	9	16	8
Baseline staff interviews	7	7	7
Follow-up staff interviews	8	7	10
Baseline student interviews	2	1	0^b^
Follow-up staff/student focus group	1	1	0
Workshop Field Notes^a^	11	9	11

^a^Supplied by more than one researcher for some events

^b^A number of attempts to organise student interviews at the rural hospital were made

*I think it has encouraged [the team], making them feel more positive about the process. The process of the teaching environment and where they fit in and what they are doing and what they are achieving and the benefits of all that. … Certainly the greater awareness of the whole clinic about - we have a main - a major teaching role here. It wasn’t just a service a clinical service delivery. It wasn’t just student coming and in and being an imposition. Now part of that - they are part of us. That is important that we give it the respect and time it needs. (Dental clinic staff member)*

*The work environment had a more social atmosphere than on the previous observation… the tutor and the dental assistances had some social conversations with the students.. Students had their own space but were rarely on their own in the work area, unlike the previous observation and the tutor was almost continuously present at a distance (work shadow observation)*

*I feel confident here, not isolated in an enclosed cubicle on my own where I can’t easily ask for help (Dental clinic student)*

As a result clinic functioning was perceived to have improved, the number of student placements was increased, patient waiting lists were reduced, and staff reported a more positive workplace culture.

Responding to the contradictions of inadequate student induction, participants at the aged care facility reported making changes in student supervision as a result of the project, such as introducing improvements to student induction and feedback.

*Have sort of recognized that we need to sort of pay a bit more attention to the student … Probably XX and I give them [students] a bit more feedback than we used to do. But we’ll actually at the end of the day ask them what they’ve done and how it went. Now we have a centralized induction on Mondays to prepare students, their documentation and learning plans. Students can now say to the Care Manager ‘this is what I need to do’ (Aged care facility staff member)*

Concerns regarding the number and structure of placements, the tensions between student log book requirements and clinical service expectations, and the opportunities for cross-disciplinary teaching were discussed but not addressed, and no changes to service delivery were implemented. The observers noted barriers to change that were not articulated by participants, including low management participation or tangible support for change. The workplace culture, while largely positive towards resident health care was quite fixed in regard to staff roles and processes, and was not influenced by the project.

The rural hospital was the most challenging of the three sites with high overt levels of individual distress and organisational dysfunction described by the participants. Individual staff were delegated by management to participate in the original project and the resulting group had to work hard to develop cohesion. By the end of the project they had achieved this, and responding to the contradiction of the expectations for student supervision when the clinical service was at risk, they developed a student charter. However, sustainability was doubtful as no implementation plan was agreed. One practical outcome was the decision to reduce the total number of students placed at the hospital to ease the workload of staff. Patient health care and staff roles did not alter.

Further analysis mapped the progress of each of the participating health services to the stages of the expansive learning cycle, as set out below:

### Questioning/need state

Prior to the project/activity, across the three sites, there was little sustained or coherent contemplation of the ways in which each health service could improve the management of student placements.

*It’s not very often that a group of people from all different sorts of area and disciplines can get together and collaborate and discuss the issues [about student clinical placements]. I think that has been a good thing…. The people on the team need to be able to have that time together and how that is going to be done I have no idea (Rural hospital staff member)*

Although reasons for this varied, improving management of student placements was a low priority in each of the sites. Without the external impetus of the current project, it appeared unlikely that any of the three sites would have devoted time and/or energy to this issue. Once the project was underway though, all participants actively engaged in questioning the current status and clarifying the challenges they faced.

### Analysis/double bind

Once the challenges were identified, participants at each of the three sites quickly moved to a more detailed consideration of practical implications in the workplace. Student supervision was generally perceived as adding to the workload of health service staff. Although staff mostly enjoyed their teaching roles, the tension between teaching and managing clinical workload was reported as professionally frustrating.

### Modeling the new solution/breakthrough

In all teams there was some consideration and recognition of the importance of cultural change in clinical settings to increase awareness of student learning needs, together with awareness of each team member’s roles and responsibilities in meeting these needs. Supported by the project activities, each team engaged in a process of identifying new approaches and innovations in their management of student clinical placements. The project supported participants at all three sites to brainstorm new ideas, articulate particular challenges and creatively identify possible solutions.

*It wouldn’t be bad to put out a generic survey to see if as student they would be interested in having a multi-disciplinary sort of approach in getting together (Rural hospital staff member)*

*There’s something we can generate that’s ongoing. We can have a patient feedback form… that can be given to the student (Dental clinic staff member1)*

*There’s still al lot I think needs to be changed in the appointment book …I know it’s all trial and error but I think we need to look at that a little bit more (Dental clinic staff member 2)*

*There was very productive discussion about using their continuous improvement planning and institutional audits to include goals related to teaching and learning (Aged Care facility workshop field note)*

*It’s been good to have the discussion about what we could do and to see that we as workers, not management, can actually do something (Aged care facility staff member)*

However, only the dental clinic team developed an implementation plan to change the way student supervision was managed.

### Examining and testing the new model/adjustment, enrichment

The three sites began to diverge more clearly at this stage with the dental clinic testing the most significant and specific changes including reconfiguration of clinical areas, changing patient bookings and staff work rosters. At the aged care facility, participating team members had suggested the development of student welcome packs and formal inductions but could take this no further due to lack of management support. Hospital staff had developed a student charter capturing their commitment to a high quality student experience but had no accompanying implementation strategy.

### Implementing the new model/resistance

At the follow up interviews and second work place observation, the dental clinic had implemented a series of changes to enhance management of student clinical placements. There was no apparent resistance from staff or senior management who supported the proposed changes and were delighted with the outcomes. At the aged care facility, there was a strong and hierarchical bureaucracy that had little inclination to change. Staff sincerely reported a positive workplace atmosphere, and that they were passionate about aged care and student learning. However, observers from the project team at the workshops saw a lack of common understandings between management and front line staff about who could enact changes. Senior management took ownership of the proposed initiatives with no associated agency for any relevant staff to implement change.

*Mostly taken over by organisation [i.e. management] apparently disempowering the team (Aged care facility workshop field note)*

Conflict was more overt at the hospital, where there was an atmosphere of crisis. Here, the project participants wanted to form a team that could make improvements to student placements, but the barriers to implementation were formidable.

*I can see the need for a team. I never saw that before. We put in place the steps to build a team, but are we committed to spend extra time?...... We have to run it; it will be disappointing if we don’t. We’re not encouraged by management. (Rural hospital staff member)*

Although developed with much passion and enthusiasm by staff, without a mechanism for sustainability within the organisation the student charter could not progress beyond a plan.

### Reflecting on the process/stabilisation and consolidating and generalising the new practice

At the conclusion of the project only the dental clinic was sufficiently progressed along a path of change to be in a position to reflect and consolidate their new practices. Debriefing participants in each of the three teams through the final focus group interviews was a positive experience for the dental clinic staff who could acknowledge and celebrate their success. By contrast members of the other two teams expressed some frustration and disappointment at an opportunity lost.

*I think locally it has worked but connecting with the others I don’t it has made any difference. (Aged care facility staff member)*

*From the outset, executive management didn’t buy into it; it was sidelined. They didn’t realise the value that could come from it. (Rural hospital staff member)*

In failing to ‘transform’ the object of their activity and to enact new ways of working, staff at the aged care facility and the rural hospital were clearly disappointed. In the case of the rural hospital it was not possible to gather the participants for a final focus group, possibly in part due to this disappointment. Instead individual interviews were conducted.

In the early phases of Building Teams for Quality Learning project at the dental clinic, patient care activity systems operated apparently independently of student supervision activity systems. When service pressures intensified, patient care imperatives out ranked student-learning needs. At the conclusion of the project, participating staff at the dental clinic had identified a new solution that transformed their approach to student supervision. This solution saw much greater integration of students into the day-to-day activities of patient care with benefits for staff, students and patients [17, 19]. Responding to identified contradictions, participating dental clinic staff had successfully transformed the object of their daily activities from patient care or student supervision, to one where these were integrated into a more combined object, and in so doing recreated their activity systems.

In a similar way to the dental clinic, in the early phases of the project, at the aged care facility and the rural hospital patient care activity systems and student supervision systems operated along side each other. As with the dental team, contradictions and tensions were observed as the staff members attempted to reconcile the apparently competing activities of high quality patient/resident care and managing students on clinical placements. Although a number of good ideas were discussed at aged care facility and the rural hospital workshops, participants in these other two health services could not develop a successful implementation plan. The key expansive learning cycle element of modeling and testing new solutions was not successfully achieved and participants were unable to collectively reassess and reinterpret the object of their activities. Without a transformed or expanded object, no new activity systems could emerge. No changes were made to the nature of interactions between people, the organisation of work tasks or in relationships with the wider community of participants. Without these foundational elements shifting, no effective change in activity systems was possible.

In the dental clinic, the patient booking system functioned as a successful boundary object. As a stable object with meaning in multiple activity systems, the booking system assisted ‘boundary crossing’ between different activity systems by participants [19, 23, 30]. It directly mediated a solution to the second identified contradiction. A similarly effective enabler of boundary crossing in the other two health services was not identified which may have been significant given the overall outcome. Workplace observations and interviews with participants at the aged care facility suggested that the student logbooks could have functioned as a potential boundary object. These log books articulated required student learning outcomes that translated directly into clinical work activities and were frequently referenced by participating staff members. However, this link was not exploited to more directly connect students with patient care within a new activity system. The situation was even more complex at the rural hospital and no stable boundary object could be identified. The suggested student charter was a potential boundary object that could have been effective in supporting change.

## Discussion

It has been noted that it is hard to change team and organisational culture in health care, especially with an external intervention [31]. While there are many papers published on how to change organisational culture in health care, there is very little research on organisational or systems approaches to health service interventions to improve student clinical placements such as the one described in this paper. Reports of the success or otherwise of student clinical placements typically describe activities and interventions by educational institutions, individual supervisors or preceptors, or students rather than the health services themselves [9, 32–35]. As such they are focused on how work based learning occurs rather than on system level changes within health services. The variability, fluidity and unpredictability of clinical practice and constant change within health services contribute to the difficulty of generalising findings of individual studies. These features of health service operations underline the importance of concepts such as the boundary objects described in activity theory in achieving successful change in many contexts. Recent work has also demonstrated the utility of activity theory and expansive learning in creating a better understanding of factors associated with organisational readiness for change, and highlighted the importance of identifying and working with contradictions [36].

Research that documents organisational change attempts in health services that have failed is also scant. This is despite recognition of the value of ‘learning from failure’ in addition to learning from successful interventions [37]. The purpose of Building Teams for Quality Learning was to build capacity for collective action among a group of health professionals. The particular focus of the initiative was to enable staff to identify and address local issues impacting on the management of student clinical placements. All participants were individually supportive of and committed to, high quality student learning experiences. However, the extent to which each team realised their aspirations for managing student clinical placements differed in each of the three groups of participants.

There are some important limitations to note in relation to this analysis. An apparent lack of successful change at the aged care facility and the rural hospital could be attributed to more complex working environments and/or a more entrenched culture of fixed staff roles and management approaches. It is to be expected also that more complex organisations will have more activity systems, many of which will be closely inter-related, and therefore will require more complex expansive learning cycles, or more cycles depending on the nature of the changes that are made. It is also the case that the intervention only really succeeded in the smallest of the three settings. Larger organisational size is usually associated with greater complexity, and it may be that in the larger organisations, the Building Teams for Quality Learning interventions were stifled by the inherently more bureaucratic and/or hierarchical workplace cultures. It is of note that the two larger organisations experienced less change due to two completely different reasons, one because of rigidity (the aged care facility), and one because of chaotic dysfunction (the country hospital). This particular observation lends itself to further investigation and research.

Although the period of engagement at each site by the project team was nearly a year from initial recruitment to final interviews, a longer period of engagement may have captured additional evidence of change to existing activity systems. Similarly, more frequent or longer periods of workplace observation may have yielded more evidence. It is also possible that the actual project intervention was less effective in these more complex organisations, a hypothesis that can be tested with implementation a wider range of health service settings.

A key aspect of the early phases of the expansive learning cycle is the coming together of individuals in a collective manner around a common desire for change. At the conclusion of the project, participants at the dental clinic emerged as a more cohesive network, while particularly at the rural hospital this outcome was not achieved. The dental clinic participants could have been assisted in this process by a clear professional imperative around dental practice and the formation of future dentists. In contrast the disciplinary profile of the aged care and hospital participants was far more disparate and multiprofessional which may have contributed to a lack of disciplinary drivers regarding the learning experiences of the students. An alternate explanation is that while most staff at the dental clinic were also participants in the project activities, only some staff from each of the other two larger sites participated in the project. In this case the dental clinic staff were already primed to become an effective network for implementing change in their activity systems. Participants at the other two sites did not come into the project with similar levels of cohesion. In the case of the rural hospital, as staff were co-opted for participation without particular regard to existing working relationships, little evidence of a cohesive team could be seen even by the end of the project.

Finally it should be noted that the focus of the original project was on building capacity in staff around the management of student clinical placements rather than on documenting or improving student learning outcomes and student learning experiences. As such there was minimal information regarding the impact of the project on student learning. Similarly there was minimal information collected on health outcomes for patients and residents of the participating health services as this was beyond the project scope. Both these considerations are of considerable importance and in the view of the project team merit specifically designed research projects.

## Conclusions

The application of activity theory and the expansive learning cycle assisted a deeper understanding of the different project outcomes with three different groups of participants. This included identifying specific points in the expansive learning cycle at which the three groups diverged. Unlike the participants at the dental clinic, participants at the aged care facility and the rural hospital were unable to focus on breakthrough thinking and to model new solutions to their dilemma of managing student clinical placements at the same time as meeting patient and resident health care needs. Successful change to activity systems was associated with a pre existing cohesive network of people who shared a common motivation for change, a supportive process that facilitated formulation and testing of new activity models, and the existence and use of boundary objects.

Practical recommendations arising from this work suggest that health services that host student clinical placements should actively seek out and embrace contradictions. These inherent tensions should be viewed as positive opportunities for enabling effective change rather than insoluble problems. Time should be spent exploring alternate models for managing student clinical placements and trialing them. Finally, stable boundary objects should be sought and used (or developed if need be) to assist and support change.
